# Comprehensive investigation of sources of misclassification errors in routine HIV testing in Zimbabwe

**DOI:** 10.1002/jia2.25700

**Published:** 2021-04-21

**Authors:** Simon Gregson, Louisa Moorhouse, Tawanda Dadirai, Haynes Sheppard, Justin Mayini, Nadine Beckmann, Morten Skovdal, Janet Dzangare, Brian Moyo, Rufurwokuda Maswera, Benjamin A Pinsky, Sungano Mharakurwa, Ian Francis, Owen Mugurungi, Constance Nyamukapa

**Affiliations:** ^1^ Department of Infectious Disease Epidemiology Imperial College London School of Public Health London UK; ^2^ Biomedical Research and Training Institute Harare Zimbabwe; ^3^ Global Solutions for Infectious Diseases San Francisco CA USA; ^4^ University of Roehampton London UK; ^5^ University of Copenhagen Copenhagen Denmark; ^6^ Zimbabwe Ministry of Health and Child Care Harare Zimbabwe; ^7^ Stanford University Stanford CA USA; ^8^ Africa University Mutare Zimbabwe

**Keywords:** HIV testing, rapid diagnostic tests, misclassification errors, false‐HIV‐negative results, false‐HIV‐positive results, Zimbabwe

## Abstract

**Introduction:**

Misclassification errors have been reported in rapid diagnostic HIV tests (RDTs) in sub‐Saharan African countries. These errors can lead to missed opportunities for prevention‐of‐mother‐to‐child‐transmission (PMTCT), early infant diagnosis and adult HIV‐prevention, unnecessary lifelong antiretroviral treatment (ART) and wasted resources. Few national estimates or systematic quantifications of sources of errors have been produced. We conducted a comprehensive assessment of possible sources of misclassification errors in routine HIV testing in Zimbabwe.

**Methods:**

RDT‐based HIV test results were extracted from routine PMTCT programme records at 62 sites during national antenatal HIV surveillance in 2017. Positive‐ (PPA) and negative‐percent agreement (NPA) for HIV RDT results and the false‐HIV‐positivity rate for people with previous HIV‐positive results (“known‐positives”) were calculated using results from external quality assurance testing done for HIV surveillance purposes. Data on indicators of quality management systems, RDT kit performance under local climatic conditions and user/clerical errors were collected using HIV surveillance forms, data‐loggers and a Smartphone camera application (7 sites). Proportions of cases with errors were compared for tests done in the presence/absence of potential sources of errors.

**Results:**

NPA was 99.9% for both pregnant women (N = 17224) and male partners (N = 2173). PPA was 90.0% (N = 1187) and 93.4% (N = 136) for women and men respectively. 3.5% (N = 1921) of known‐positive individuals on ART were HIV negative. Humidity and temperature exceeding manufacturers’ recommendations, particularly in storerooms (88.6% and 97.3% respectively), and premature readings of RDT output (56.0%) were common. False‐HIV‐negative cases, including interpretation errors, occurred despite staff training and good algorithm compliance, and were not reduced by existing external or internal quality assurance procedures. PPA was lower when testing room humidity exceeded 60% (88.0% vs. 93.3%; *p* = 0.007).

**Conclusions:**

False‐HIV‐negative results were still common in Zimbabwe in 2017 and could be reduced with HIV testing algorithms that use RDTs with higher sensitivity under real‐world conditions and greater practicality under busy clinic conditions, and by strengthening proficiency testing procedures in external quality assurance systems. New false‐HIV‐positive RDT results were infrequent but earlier errors in testing may have resulted in large numbers of uninfected individuals being on ART.

## 1 INTRODUCTION

Rapid HIV diagnostic tests (RDTs) have high sensitivity and specificity under controlled laboratory conditions [1, 2]. However, there is growing evidence that RDT‐based HIV‐testing algorithms can provide incorrect results [3, 4] (misclassification errors) when used in routine health services. In a comparison of RDT results from prevention of mother‐to‐child transmission (PMTCT) programme records with results from quality assurance testing conducted at central laboratories, negative‐percent agreement (NPA; the percent of true HIV‐negative cases with negative RDT results) and positive‐percent agreement (PPA; the percent of true HIV‐positive cases with positive RDT results) ranged from 98.5% to 99.9% and 76% to 98%, respectively, across nine countries [4, 5, 6]. Errors in quality assurance testing may contribute to these findings and, a World Health Organisation (WHO)‐led a systematic review of peer‐reviewed articles, abstracts and grey literature published in 2017 found lower proportions of false‐HIV‐positive (median 3.1%, inter‐quartile range (IQR): 0.4% to 5.2%) and false‐HIV‐negative (median 0.4%: IQR: 0% to 3.9%) diagnoses [3]. However, the large numbers of people tested using RDT‐based algorithms in routine services in sub‐Saharan Africa (56.5 million in 2014) mean that, even with these lower levels of errors, as many as 93,000 people could be misdiagnosed annually [7]. This is important because false‐HIV‐negative results can lead to failure to provide ART, PMTCT and early infant diagnosis (EID) services, and to use HIV prevention services, which can cause increased morbidity, mortality and new infections [8, 9]. Equally, false‐HIV‐positive test results can lead to inappropriate ART initiation, causing unnecessary side‐effects, stigma and psychological distress [10]. Addressing misclassification errors in routine HIV testing, therefore, is central to meeting the global goal to end AIDS as a public health threat by 2030 [11].

Several sources of misclassification errors have been reported, including suboptimal testing strategies, weak reactive results, user error, clerical error, poor management and supervision systems, cross‐reactivity, acute/early infection and re‐testing people on ART [3]. However, their contributions have not been quantified and compared systematically. The objectives of this study were to provide in‐depth data on levels and a range of different possible sources of misclassification errors arising from the use of an RDT‐based HIV testing algorithm in health services in a sub‐Saharan African country with a generalized HIV epidemic. The study used data from routine PMTCT services in Zimbabwe extracted in the 2017 round of national antenatal (ANC) HIV surveillance and external HIV testing quality assurance data from central laboratories done to evaluate HIV surveillance estimates. The HIV surveillance was extended to collect data on (i) effectiveness of active quality management systems, (ii) RDT kit performance under local environmental conditions, (iii) user and clerical errors, (iv) reliability of clinic data on “known‐positive” cases (i.e. individuals previously diagnosed HIV‐positive according to clinic records), and (iv) the wider generalizability of findings for pregnant women.

## 2 METHODS

### 2.1 Data sources

National ANC HIV surveillance was done using routine PMTCT programme data in 62 sites, selected to represent Zimbabwe’s ten provinces, and ran from April 1 to September 30, 2017 [12]. HIV surveillance methods followed WHO guidelines [12] with individual client data being extracted onto ANC surveillance forms and submitted to a central team in Harare for analysis. Full details of the surveillance procedures have been published [13].

To measure misclassification errors in RDT algorithms, dried blood spot (DBS) specimens were collected at all participating clinics and transported to Harare for laboratory testing. Procedures were added to the ANC HIV surveillance to measure possible sources of misclassification errors and effectiveness of quality assurance and training programmes. To investigate RDT performance under local environmental conditions, trained nursing staff placed data loggers [14] in testing areas and storerooms at all ANC surveillance sites to measure whether the temperature and humidity in these areas exceeded the manufacturer’s recommended limits (maximum temperature, 30^0^ centigrade; and maximum humidity, 60%; recommended for the Determine™ HIV‐1/2 screening test [15]). Instances where weak reactive results, heavily shaded backgrounds, or other problems made RDT outputs difficult to interpret were captured on the ANC surveillance forms. To measure the frequency of cases with unclear output, trained nursing staff at a convenience sample of seven surveillance sites in Manicaland province used a Smartphone camera application [16] to capture and transmit images of RDT outputs for interpretation by two independent experts in laboratory diagnostics (IF and HS). To investigate cross‐reactivity, tests to detect coinfections (syphilis and malaria) that could contribute to misdiagnosis in RDT testing [17] were conducted using DBS specimens collected from HIV surveillance participants.

The Smartphone camera application was also used to capture the time elapsed between test initiation and reading results (15 minutes recommended for the Determine™ HIV‐1/2 test [15]), and information from ANC surveillance forms so that the frequencies of other types of user error (interpretation errors) and clerical error (recording errors) could be measured. RDT expiry dates were captured on the ANC surveillance form.

**Figure 1:**
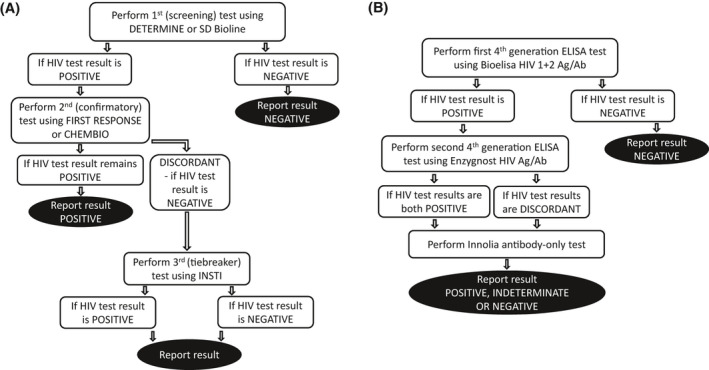
HIV testing algorithms used in Zimbabwe in 2017 at the time of the national HIV surveillance round. **(A)**. National algorithm used in routine healthcare services. **(B)**. Final algorithm used by the central laboratories to provide external quality assurance for HIV surveillance

Coverage and effectiveness of national quality management systems for routine HIV testing were assessed using site‐level meta‐data on local implementation of internal (IQA) and external (EQA) quality assurance procedures, collected on a site assessment form; and individual‐level data on training received by staff conducting HIV tests, compliance with the national HIV testing algorithm (Figure 1A), and compliance with guidelines to avoid re‐testing individuals already on ART, collected on the surveillance form.

Pregnant women’s male partners who attended ANC for couple HIV testing and counselling were included in the surveillance so that the generalizability of the results for pregnant women could be explored.

### 2.2 Laboratory methods

#### 2.2.1 HIV testing quality assurance procedures done for HIV surveillance

Linked laboratory‐based HIV testing using the DBS specimens was done for quality assurance and to provide a gold standard to evaluate bias in HIV estimates and trends due to changes in surveillance procedures. All HIV surveillance participants were eligible for quality assurance testing. This testing was done at Zimbabwe’s National Microbiology Reference Laboratory (NMRL) (ISO accredited MED009) for women and at the Biomedical Research and Training Institute’s (BRTI; https://www.brti.co.zw/) laboratory for men (ISO accredited MED020). The original quality assurance algorithm specified an initial fourth‐generation screening ELISA test (Bioelisa HIV 1 + 2 Ag/Ab) [18], a second fourth‐generation confirmatory ELISA test (Enzygnost HIV Ag/Ab) [19] to confirm positive results, and an INNO‐LIA^TM^ HIV‐I/II (INNO‐LIA) antibody test [20] to resolve discrepant results. Later, the algorithm was modified to include INNO‐LIA antibody‐only testing for *all* Bioelisa‐positive cases to provide a consistent basis for comparison with the antibody‐based RDT results (Figure 1B). NMRL evaluated, validated and optimized the laboratory HIV test kits for use on DBS specimens before they were used in the study.

#### 2.2.2 Pro‐viral DNA HIV diagnostic tests

Unexpectedly high proportions of cases were found where the Enzygnost ELISA result was HIV‐positive but the INNO‐LIA result was negative (women: 21.0%, N = 3707; men: 2.6%, N = 340). There were also high proportions of individuals with HIV‐positive results in previous testing (“known‐positive” cases) with HIV‐negative quality assurance results (women: 10.0%, N = 1835; men: 9.0%; N = 190). To develop a more conclusive gold standard for HIV status, a GeneXpert qualitative pro‐viral DNA test [21] was run on samples of cases with discordant and concordant RDT, ELISA and INNO‐LIA results. The DNA test has a high sensitivity and a low limit of detection for HIV infection in people living with HIV on ART with undetectable viral load (B. A. Pinsky et al. 2019, personal communication). Details of these tests are given in Supporting Information.

#### 2.2.3 Syphilis and malaria tests

Details of laboratory methods used for syphilis and malaria detection are given in Supporting Information.

### 2.3 Statistical analysis

RDT misclassification errors were assessed using: (1) PPA and NPA statistics, and (2) the proportion of true HIV‐positive cases amongst “known‐positive” individuals. Two estimates were produced for each statistic: the first using a gold standard based on results from the laboratory HIV‐testing quality assurance algorithm (Figure 1B); and the second using the gold standard adjusted for the DNA test results. In making these adjustments, it was assumed that, for each combination of antenatal RDT test (or “known‐positive” clinic record status) and external laboratory quality assurance ELISA and INNO‐LIA test results, the proportion with errors found in the sample of cases tested with the DNA test was representative of the proportion for all cases with the same pattern of antenatal and laboratory results. Further details are available in Supporting Information.

PPA and NPA estimates (with 95% confidence intervals [95%CIs]) based on the adjusted gold standard were calculated and compared between pregnant women and male partners of pregnant women; and by age‐group, location (province and urban/peri‐urban/rural), testing with or without the male partner and site type (main site [district hospital] vs. sub‐site [satellite clinic] in the national HIV surveillance).

Indicators of weak quality management systems (absence of IQA and EQA procedures, staff not trained in RDTs, and non‐compliance with national HIV testing algorithm (Figure 1A)), poor RDT kit performance under local conditions (temperature and humidity in testing rooms and storerooms exceeding manufacturers’ recommendations), and user and clerical errors (screening test expired or screening test output read too soon) were calculated to investigate the contributions of different possible sources of RDT misclassification errors identified in the literature [3]. To assess the contributions of these different sources of error, PPA and NPA estimates (with 95%CIs), using the adjusted gold standard, were calculated and compared for RDTs done in the presence and absence of each indicator.

Estimates were calculated for the sensitivity of the laboratory (Bioelisa HIV 1 + 2 and Enzygnost Ag/Ab ELISAs and INNO‐LIA) and rapid diagnostic tests (Determine^TM^ HIV 1/2 and Chembio HIV 1/2 STAT‐PAK®) in detecting HIV infection in “known positive” pregnant women on ART by using the adjusted gold standard results.

Data analysis was conducted using Stata version 14.

Ethical approval was granted by the Medical Research Council of Zimbabwe (MRCZ/A/1965) and the Imperial College Research Ethics Committee (15IC2797). Study participants provided written informed consent for routine PMTCT HIV testing and for remnants of a blood sample taken for routine ANC testing to be used in quality assurance testing. In Zimbabwe, pregnant women aged 15 years are considered emancipated minors and are able to provide consent. All women and men eligible for the study provided informed consent.

## 3 RESULTS

### 3.1 Sample characteristics

**Figure 2:**
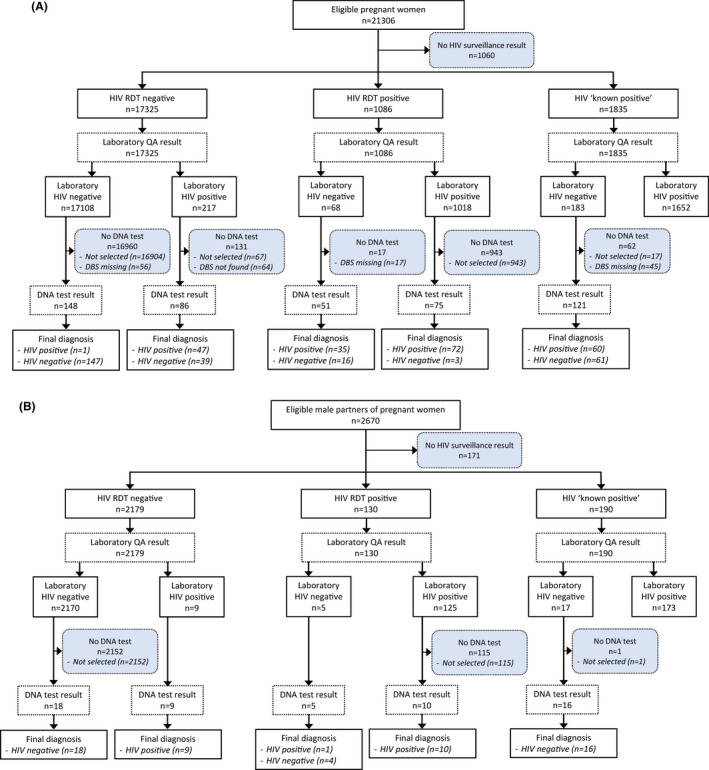
STARD 2015 flow diagram. (A). Pregnant women. (B). Male partners of pregnant women

21,306 women were eligible and consented to participate in the national HIV ANC surveillance – 20,246 (95.0%) had HIV RDT results (N = 18,411) or were “known‐positive” cases (N = 1835) (Figure 2A). Corresponding numbers for men were 2670, 2499 (93.6%), 2309 and 190 (Figure 2B). HIV prevalence estimates based on the RDT and initial laboratory quality‐assurance results were 14.25% (95%CI, 13.8% to 14.7%) for women and 12.1% (10.8% to 13.4%) for men [13]. Table 1 shows the socio‐demographic characteristics of surveillance participants by HIV‐infection status. Determine™ HIV‐1/2 and First Response were used as the screening and confirmatory RDTs in 99.3% (N = 18540) and 98.3% (N = 1052) cases respectively. Chembio, rather than INSTI, was used as the tiebreaker RDT and gave an HIV‐positive result in 4/11 cases (no tiebreaker result was recorded for 15 other cases with discordant screening/confirmatory results).

**Table 1:** Characteristics of pregnant women and male partners participating in HIV surveillance, Zimbabwe, 2017, by HIV infection status based on laboratory testing

Characteristic	Women				Men			
	HIV‐positive		HIV‐negative		HIV‐positive		HIV‐negative	
	n	%	n	%	n	%	n	%
N	1235	100.0	17176	100.0	134	100.0	2175	100.0
Province								
Bulawayo	28	2.3	515	3.0	1	0.7	52	2.4
Harare	122	9.9	2164	12.6	7	5.2	180	8.3
Manicaland	133	10.8	1895	11.0	13	9.7	230	10.6
Mashonaland Central	110	8.9	1931	11.2	11	8.2	239	11.0
Mashonaland East	160	13.0	2440	14.2	22	16.4	374	17.2
Mashonaland West	131	10.6	1598	9.3	16	11.9	236	10.9
Masvingo	109	8.8	1848	10.8	18	13.4	234	10.8
Matabeleland North	127	10.3	1527	8.9	14	10.4	188	8.6
Matabeleland South	175	14.2	1468	8.5	10	7.5	208	9.6
Midlands	140	11.3	1790	10.4	22	16.4	234	10.8
Geographic areas								
Urban	471	38.1	6966	40.6	58	43.3	884	40.6
Peri‐urban	76	6.2	1034	6.0	8	6.0	131	6.0
Rural	678	54.9	9086	52.9	68	50.7	1153	53.0
Not recorded	10	0.8	90	0.5	0	0.0	7	0.3
Site type								
Main site	639	51.7	9063	52.8	89	66.4	1495	68.7
Sub‐site	298	24.1	4280	24.9	45	33.6	680	31.3
Not recorded	298	24.1	3833	22.3	0	0.0	0	0.0
Age‐group								
15 to 19 years	179	14.5	3665	21.3	1	0.7	49	2.3
20 to 24 years	353	28.6	5338	31.1	14	10.4	492	22.6
25 to 34 years	544	44.0	6460	37.6	72	53.7	1101	50.6
35 to 49 years	142	11.5	1555	9.1	43	32.1	488	22.4
≥50 years	0	0.0	0	0.0	3	2.2	35	1.6
Not recorded	17	1.4	158	0.9	1	0.7	10	0.5
Marital status								
Single	120	9.7	1021	5.9	–		–	
Married	1076	87.1	15936	92.8				
Formerly married	28	2.3	139	0.8				
Not recorded	11	0.9	80	0.5				
Education level								
Primary or none	303	24.5	3354	19.5	28	20.9	305	14.0
Secondary or above	921	74.6	13746	80.0	104	77.6	1856	85.3
Not recorded	11	0.9	76	0.4	2	1.5	14	0.6
Pregnancy trimester								
First	318	25.7	4050	23.6	–		–	
Second	676	54.7	9606	55.9				
Third	227	18.4	3352	19.5				
Not recorded	14	1.1	168	1.0				

### 3.2 Levels of HIV misdiagnosis

**Figure 3:**
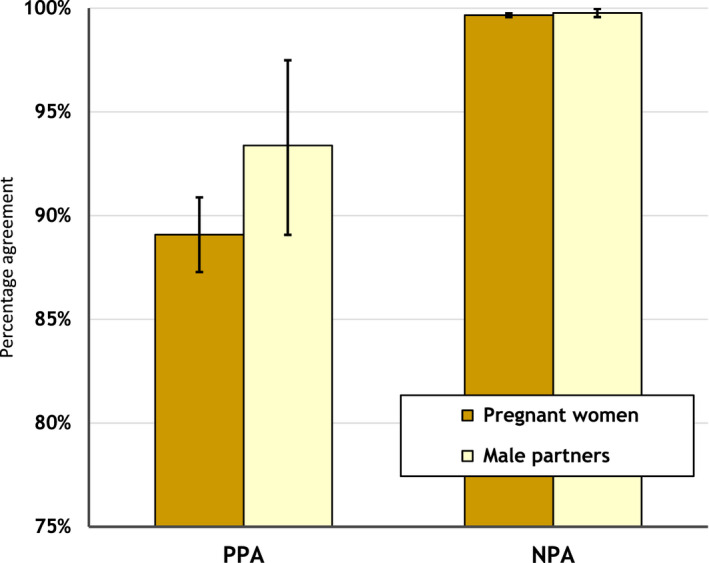
Positive percent agreement (PPA) and negative percent agreement (NPA) for HIV rapid diagnostic test results for pregnant women and male partners obtained in the routine prevention of mother‐to‐child transmission services compared with a gold standard derived from a laboratory‐based quality assurance HIV testing algorithm adjusted using results from a qualitative pro‐viral DNA HIV diagnostic test, Zimbabwe, 2017

PPA and NPA for women were 82.4% (95%CI, 80.2% to 84.5%; N = 1235) and 99.6% (99.5% to 99.7%; N = 17176), respectively, based on the initial laboratory gold standard. Corresponding results for men were 93.3% (87.6% to 96.9%; N = 134) and 99.8% (99.5% to 99.9%; N = 2175). Using the DNA test‐adjusted gold standard (Figure 3), PPA and NPA improved to 89.1% (87.3% to 90.9%) and 99.7% (99.6% to 99.8%) for women (Table 2), and 93.4% (89.2% to 97.6%) and 99.86% (99.6% to 100%) for men (Table S5).

**Table 2:** Positive percent agreement^a^ for HIV rapid diagnostic tests in pregnant women by socio‐demographic characteristic

Characteristic	% (95%CI)	n	N
All pregnant women	89.1 (87.3 to 90.9)	1028	1154
Province			
Bulawayo	89.3 (78.1 to 100)	26	29
Harare	87.7 (81.9 to 93.5)	108	123
Manicaland	89.7 (84.4 to 95.0)	114	127
Mashonaland Central	89.5 (83.8 to 95.3)	97	108
Mashonaland East	87.4 (81.6 to 93.1)	112	128
Mashonaland West	87.8 (82.1 to 93.5)	112	128
Masvingo	96.1 (92.3 to 99.9)	95	99
Matabeleland North	94.1 (89.8 to 98.3)	111	118
Matabeleland South	88.5 (83.4 to 93.6)	135	152
Midlands	89.2 (83.9 to 94.6)	116	130
Geographical area			
Urban	92.5 (90.0 to 94.9)	420	454
Peri‐urban	89.7 (82.2 to 97.1)	58	64
Rural	84.9 (82.1 to 87.7)	539	636
Site type			
Main site	90.3 (88.0 to 92.7)	547	606
Sub‐site	88.5 (84.6 to 92.4)	229	258
Age‐group			
15 to 19 years	89.8 (85.1 to 94.5)	144	160
20 to 24 years	90.6 (87.4 to 93.7)	303	334
25 to 34 years	90.5 (87.9 to 93.2)	433	479
35 to 49 years	88.9 (83.8 to 94.0)	130	146
Single or couple testing			
Single	89.5 (87.7 to 91.4)	918	1026
Couple	93.2 (88.7 to 97.8)	111	119

^a^ Using the revised gold standard for the presence of HIV infection based on the HIV surveillance laboratory quality assurance results after adjustment for the results from the qualitative pro‐viral DNA tests. DNA tests.

### 3.3 Sources of HIV RDT misclassification errors

Table 3 compares the adjusted PPAs for factors that could contribute to false‐negative HIV test results.

**Table 3:** Positive percent agreement for HIV RDTs in pregnant women by presence and absence of environmental, quality management, user‐error, and cross‐reactivity factors

Factor	Factor present			Factor absent			Proportion with characteristic
	% (95%CI)	n	N_1_	% (95%CI)	n	N_2_	N_1_/(N_1_ + N_2)_
Weak quality management systems							
Lack of quality assurance							
No internal quality assurance	96.0 (91.6 to 100)	72	75	89.2 (87.3 to 91.1)	927	1039	0.067
No external quality assurance	90.8 (88.8 to 92.9)	699	770	86.8 (82.8 to 90.9)	233	268	0.742
Lack of staff training							
External training not received in all kits used	93.4 (87.5 to 99.3)	64	68	89.0 (87.1 to 91.0)	892	1001	0.064
Not all staff trained in screening test	93.6 (90.1 to 97.2)	170	182	89.0 (86.1 to 91.9)	391	439	0.293
Non‐compliance with national algorithm	96.5 (92.5 to 100)	82	85	89.5 (87.6 to 91.3)	945	1056	0.074
Poor RDT performance under local conditions							
Screening test band unclear	93.3 (80.7 to 100)	14	15	89.9 (88.1 to 91.6)	1001	1114	0.013
Temperature above recommendation (>30°)							
Testing room	94.7 (84.7 to 100)	18	19	89.2 (87.2 to 91.2)	822	921	0.020
Store room	90.2 (88.3 to 92.1)	819	908	93.0 (88.3 to 97.7)	106	114	0.889
Humidity above recommendation (>60%)							
Testing room	87.7 (84.9 to 90.6)	451	514	92.7 (90.2 to 95.2)	396	427	0.546
Store room	90.8 (89.0 to 92.6)	907	999	87.9 (75.1 to 100)	22	25	0.976
Biological cross‐reactivity							
Syphilis history (TPHA+)	100.0 (‐)	55	55	90.4 (88.6 to 92.3)	888	982	0.053
Pregnancy stage (1st trimester)	87.3 (83.4 to 91.2)	244	279	90.8 (88.8 to 92.7)	771	850	0.247
User and clerical errors							
Screening test expired	90.7 (82.6 to 98.9)	44	49	89.9 (88.1 to 91.6)	985	1096	0.042
Screening test reading taken too soon (<15 m)	97.6 (92.8 to 100)	40	41	88.6 (78.0 to 99.1)	31	35	0.539

Results based on the total of 1154 HIV‐positive pregnant women after corrections based on the qualitative pro‐viral DNA test results.

For indicators of weak quality management systems, routine EQA was implemented in a quarter of cases but did not improve PPA. Staff training and IQA levels were high, and relatively few cases (7.4%) of non‐compliance with the national HIV testing algorithm were recorded.

For indicators of RDT kit performance, nursing staff reported few cases where the screening test produced an unclear line (1.3%). The independent assessors reported unclear RDT output in 3.7% (47/1258) of cases in the sites where the Smartphone camera application was used. Testing room temperatures rarely exceeded manufacturers’ recommended maximums (2.0% of pregnant women); but these limits were often exceeded in the storeroom (88.9%) and, for humidity, in the testing room (54.6%) and the storeroom (97.6%). A lower PPA was recorded when testing room humidity exceeded the recommended maximum (87.7% [84.9% to 90.6%] vs. 92.7% [90.2% to 95.2%]). PPA was not reduced by syphilis history, malaria infection, or in the first trimester of pregnancy. Malaria infection was found in 2.9% (2/69) and 6.25% (5/80) of HIV false‐negative and concordant‐positive cases respectively (χ^2^ = 0.93; *p* = 0.3).

For indicators of user and clerical errors, expired test kits were used in 4.2% of cases. In sites with the Smartphone camera application, premature readings of screening test output were observed in 53.9% (41/76) of cases but the PPA was not reduced in these cases. No cases interpreted as HIV‐negative by the independent assessors were recorded as HIV‐positive by clinic staff (N = 1337). However, 2.9% (4/139) of cases interpreted as HIV‐positive by the independent assessors were recorded as HIV‐negative by the clinic staff; these cases may be interpretation errors as no confirmatory test result was recorded. In three cases (one interpreted as HIV‐positive and two as HIV‐negative by the clinic staff), the independent assessors interpreted the RDT output as invalid due to poor kit application or performance. In one of 13 cases where the ELISA, INNO‐LIA and DNA tests produced consistent HIV‐positive results, the clinic staff and the independent assessors both read the RDT result as HIV‐negative; suggesting a failure of the Determine™ HIV‐1/2 test kit.

NPA was lower in women with a history of syphilis (94.3% [91.4% to 97.3%] vs. 99.7% [99.7% to 99.8%]) and when some staff at the clinic had not received training in the screening test (99.2% [98.9% to 99.5%] vs. 99.9% [99.9% to 100%]). No other factors showed associations with reduced NPA (Table S6).

### 3.4 Levels and patterns of past mis‐diagnosis of HIV infection in patients on ART

The proportion of “known‐positive” cases confirmed as HIV‐positive in the initial laboratory tests was 90.0% (95%CI, 88.6% to 91.4%) for women and 91.1% (86.1% to 94.7%) for men. Using the DNA test‐adjusted gold standard, this proportion increased to 95.0% for women but remained unchanged for men (Table S4). The adjusted proportion confirmed as HIV‐positive was 96.5% (N = 1921) for “known‐positive” women and men (combined) on ART and 40.0% (N = 10) for those not on ART.

In laboratory testing of “known‐positive” pregnant women on ART, in the DNA test‐adjusted results, the ELISA tests had a sensitivity of 97.7% (1693/1733), the INNO‐LIA test had a sensitivity of 97.3% (1648/1693) in ELISA‐positive cases, and the Determine^TM^ HIV 1/2 and Chembio HIV 1/2 STAT‐PAK® RDTs had sensitivities of 84.8% (59/69) and 95.5% (66/69) respectively; indicating that, in most cases, HIV antibodies remain detectable in people‐living‐with‐HIV (PLHIV) on ART.

## 4 DISCUSSION

High proportions of HIV‐positive pregnant women (10.9%) and men (6.6%) attending routine PMTCT services in Zimbabwe in 2017 received false‐negative results. The proportions of HIV‐negative pregnant women (0.34%) and men (0.14%) receiving false‐positive results were low but 3.5% of women already on ART may be uninfected.

The high NPA compares favourably with earlier reports for Zimbabwe (2012: 98.7%) and elsewhere [3, 4, 22]. The low PPA is consistent with previous estimates for Zimbabwe [23] – although an unpublished analysis of EQA data suggested a higher PPA in 2012 (94.9% *vs*. 91.2%) [24] – and for other African countries [4]. The WHO systematic review found fewer false‐HIV‐negative results (median: 0.4%; IQR: 0.3% to 3.9%). However, most studies were small‐scale and localized, and the review may have suffered from publication bias [3]. In the review, most studies reporting false‐HIV‐negative results found suboptimal testing strategies; often using screening tests with high specificity but low sensitivity [3]. The Determine™ HIV‐1/2 test (Abbott Diagnostic Division, Hoofddorp, The Netherlands) used in Zimbabwe had a reported sensitivity of 99.4% [25]. In validated and optimized off‐label RDT runs at BRTI on DBS specimens from DNA‐confirmed HIV‐positive “known‐positive” individuals on ART, we found sensitivities of 84.8% and 95.5% for Determine™ HIV 1/2 and Chembio HIV 1/2 STATPAK® respectively. These results are consistent with reports of false‐HIV‐negative RDT results for individuals at late stages of disease or on ART [3, 26]. PLHIV on ART not identified but re‐tested in PMTCT services therefore may contribute to our low PPA estimate. False‐HIV‐negative RDT results can occur in early/acute infections, before antibodies appear, but should not contribute to the low PPA as the laboratory gold standard included an antibody‐only test (INNO‐LIA).

No predominant source of false‐HIV‐negative results was identified. However, several possible contributing factors were common including humidity and temperature levels in clinic testing rooms and storerooms that exceeded manufacturers’ recommendations and RDT output readings taken earlier than recommended. PPA was reduced when testing room humidity exceeded 60% (87.7% *vs*. 92.7%; *p* = 0.016). RDT output was misinterpreted in some instances. Misclassification errors occurred despite high coverage of staff training, and implementation of routine quality assurance procedures. In non‐facility‐based testing in South Africa, low testing sensitivity (45% to 54%) was attributed to a suboptimal algorithm, inadequate quality assurance and user error [22]; however, no effect sizes were reported.

Strengths of this study include national coverage, large sample size, high participation rates in the underlying HIV surveillance, a robust gold standard (with discrepant results confirmed with a DNA test), and independent identification of reading and interpretation errors using a Smartphone camera application. Some potential sources of misclassification errors were not evaluated including high temperature and humidity during transportation of test kits and cross‐contamination of specimens. HIV surveillance was done during Zimbabwe’s dry winter months; if high temperatures and humidity contribute to false‐HIV‐negative RDT results, these may be more common during the hot rainy season. Small sample sizes prevented measurement of errors in confirmatory tests and premature reading of RDT output and investigation of reasons for the higher PPA in men compared to pregnant women. No data on duration on ART were captured in the ANC surveillance preventing the investigation of associations between long‐term ART and loss of antibodies. Our estimate for premature readings of RDT output may be overstated if nursing staff started the Smartphone camera application after starting the test.

Several changes to international and national policy occurred after 2017. Zimbabwe’s national HIV testing algorithm was amended in 2018 to include re‐running screening and confirmatory tests when initial results are discordant, and limiting the tie‐breaker test to when results remain discordant. A repeat test to confirm HIV‐infection is done now prior to ART initiation. Further WHO guidance, released in November 2019, recommends three consecutive reactive tests to establish an HIV‐positive diagnosis [27]. These changes and strengthened EQA procedures [28] should consolidate the reductions in false‐HIV‐positive errors found here.

False‐HIV‐negative results have received less attention. Repeat testing is recommended for pregnant women tested in the first trimester and high‐risk individuals to identify window‐period infections [29]. In 17 districts of Zimbabwe, 72.7% of pregnant women were retested (EGPAF, personal communication, July 2018) which should reduce the impact of false‐HIV‐negative RDT results. However, in pregnant women, mother‐to‐child transmission can occur *in utero* before repeat testing and ART initiation. In these women and other groups, continued disease progression and unprotected sex prior to repeat testing could result in new adult infections and mortality. Switching to RDT kits with higher sensitivity under real‐world conditions (e.g. high humidity) and greater practicality under busy clinic conditions (e.g. with shorter readout periods) [30], and widening eligibility criteria for repeat testing could reduce the impact of false‐HIV‐negative results. EQA systems could be strengthened by performing root‐cause analysis [31] for false‐HIV‐negative cases found in proficiency testing, feeding back results to HIV testers (to increase their awareness of misdiagnoses [30]), and taking corrective action. Smartphone camera applications could be used in EQA to monitor RDT output and accuracy of testers’ interpretations of these outputs in samples of testing sites.

Substantial numbers (3.5%) of PLHIV on ART in Zimbabwe appear to be uninfected. In 2017, 978,000 adults were on ART [32] at an annual cost of $450 each [33]; therefore, more than $15 million may be wasted on unnecessary treatment every year. This situation may reflect past use of suboptimal testing strategies, RDT kits and procedures. Similar reports have been made previously [34] but this is the first quantification from large‐scale laboratory retesting of people on ART. If confirmed, major programmes of retesting of people on ART could be needed together with carefully conducted public relations programmes. Late disease‐stage HIV infection and ART can reduce the reliability of antibody‐ and RNA antigen‐based detection, so algorithms including a qualitative pro‐viral DNA test may be required to establish the true infection status of these individuals.

## 5 CONCLUSIONS

False‐HIV‐negative RDT results remained common in Zimbabwe in 2017 and risked missed opportunities for PMTCT, EID, and adult HIV‐prevention. Errors could be reduced with HIV testing algorithms that use RDTs with higher sensitivity under local climatic conditions and greater practicality under busy clinic conditions, and by strengthening EQA proficiency testing procedures. False‐HIV‐positive RDT results are infrequent now but many people already on ART may be uninfected. Further research is needed to assess the generalizability of these findings, evaluate recent improvements to HIV testing procedures, and establish the extent to which inadvertent re‐testing of PLHIV on ART with RDTs contributes to false‐HIV‐negative results.

## COMPLETING INTERESTS

SG declares shareholdings in pharmaceutical companies [GSK and Astra Zeneca]; all other authors have no conflicts of interest to declare.

## AUTHORS’ CONTRIBUTIONS

SG conceived and was involved in the design of the study, led the data analysis and interpretation, and drafted the manuscript. LM and TD managed the data, and assisted with the data analysis and interpretation. IF, HS, JM, BAP, SM, JD, MM, BM, RM, NB and MS contributed to the study design, and implementation and interpretation of the results. CN and OM were the senior authors involved with study conception and design, study oversight, and data interpretation and assisted with drafting the manuscript. All authors reviewed and provided critical edits to the manuscript.

## Supporting information


**Table S1**. Pro‐viral DNA test results by ANC and laboratory test concordance
**Table S2**. Estimates of Positive Percent Agreement and Negative Percent Agreement for pregnant women corrected based on qualitative pro‐viral DNA test results
**Table S3**. Estimates of Positive Percent Agreement and Negative Percent Agreement for male partners of pregnant women corrected based on qualitative pro‐viral DNA test results
**Table S4**. Misdiagnosis in people with HIV‐positive clinic records (“known‐positives”) corrected based on qualitative pro‐viral DNA test results
**Table S5**. Negative percent agreement for HIV rapid diagnostic tests in pregnant women by socio‐demographic characteristic
**Table S6**. Negative percent agreement for HIV RDTs in pregnant women by presence and absence of environmental, quality management, user‐error and cross‐reactivity factors
