# Supplementary material for: Comprehensive investigation of sources of misclassification errors in routine HIV testing in Zimbabwe
Source: J Int AIDS Soc. 2021 Apr 21;24(4):e25700. doi: 10.1002/jia2.25700 (PMC8059712; doi:10.1002/jia2.25700)
Supplement: Supplementary file 1 — Table S1. Pro‐viral DNA test results by ANC and laboratory test concordance Table S2. Estimates of Positive Percent Agreement and Negative Percent Agreement for pregnant women corrected based on qualitative pro‐viral DNA test results Table S3. Estimates of Positive Percent Agreement and Negative Percent Agreement for male partners of pregnant women corrected based on qualitative pro‐viral DNA test results Table S4. Misdiagnosis in people with HIV‐positive clinic records (“known‐positives”) corrected based on qualitative pro‐viral DNA test results Table S5. Negative percent agreement for HIV rapid diagnostic tests in pregnant women by socio‐demographic characteristic Table S6. Negative percent agreement for HIV RDTs in pregnant women by presence and absence of environmental, quality management, user‐error and cross‐reactivity factors [file JIA2-24-e25700-s001.docx]

**Comprehensive Investigation of Sources of Misclassification Errors in Routine HIV Testing in Zimbabwe**

**SUPPORTING INFORMATION**

**1. │ LABORATORY METHODS**

**Pro-viral DNA HIV diagnostic tests**

To provide a more robust gold standard for the presence of HIV infection, a pro-viral DNA diagnostic test (1) was run on GeneXpert using left-over dried blood spot (DBS) specimens collected from pregnant women and male partners for samples of individuals with discordant and concordant RDT, ELISA and Innolia test results. Random samples of cases were selected initially for each category of discordant and concordant results. However, stored DBS specimens could not be recovered for some of the selected cases so the final sample sizes were reduced in several instances. Details of the original and final sample sizes and the DNA test results for each category of initially discordant/concordant results are given in Table S1.

For women and men with concordant positive and concordant negative RDT, ELISA and Innolia test results, the DNA test results were in agreement in almost all cases. However, for women, the DNA test results were only in agreement with the original laboratory-based quality-assurance test results in approximately half of the cases where these results had differed from those obtained in routine RDT testing at antenatal clinics.

In the sample taken from the group with RDT-negative, ELISA-positive, Innolia-negative results, the DNA tests gave HIV-negative results in almost all cases (99.0%; N=102) – suggesting a high level of false-positive ELISA results. This was a large group for pregnant women (N=650) and, in some of these cases, the second ELISA test was not run as it was thought to be redundant given that the quality assurance algorithm required an Innolia test to be run for all cases with a positive screening test result. It is possible, therefore, that this contributed to the high level of false-positive ELISA results. No information is available on the overall number or the specific individual cases for which the first ELISA test result was positive but the second ELISA test was not done. However, for male partners, where the second ELISA test was done in all cases for which the result from the first ELISA test was positive, there was 100% (N=338) agreement between first and second ELISA test results. For RDT-positive cases where ELISA results were positive and Innolia results were negative, DNA test results were generally positive (95.0%, N=20).

For known-positive pregnant women at HIV surveillance clinics, the DNA test produced an HIV-positive result in close to half of the cases for which either the ELISA test or the Innolia test had yielded a negative result. For known-positive male partners, the DNA result agreed with the ELISA result in all 16 cases where the latter produced a negative HIV result.

**Sensitivity of the pro-viral DNA HIV diagnostic test when using stored DBS specimens**

In three of the 75 cases for which the DNA test was conducted in pregnant women with HIV-positive results from RDT tests done at the antenatal clinics and from both ELISA and Innolia tests done at the central laboratories, the DNA test result was HIV-negative. As the DNA tests were conducted on DBS specimens that had been stored for two years following their collection in the national HIV surveillance project, this raised the possibility that the DNA tests could be giving false-negative results in some instances. This would be important for the current study as it could lead to incorrect conclusions being drawn about the true level of known-positive cases that are actually HIV-negative.

Further investigation revealed that the DBS specimen used for one of the cases for which the DNA test gave an apparently false-negative result had a low volume and was of poor quality. We therefore carried out a visual inspection of the 118 DBS specimens from which the DNA test had produced an HIV-negative result when the results from the routine health service tests (including known-positive cases) and/or the laboratory tests had been positive. In all cases, the DBS specimens appeared to be in good condition.

**Reliability of HIV RDTs for people living with HIV on antiretroviral treatment**

The quality assurance data from the current study (including the DNA test results) indicate that sizeable fractions of known-positive individuals may have been misdiagnosed in the past as having HIV-infection. In addition, in the 2017 round of national HIV antenatal clinic surveillance in Zimbabwe, 7 known-positive pregnant women (and confirmed HIV-positive with the DNA test) recorded in the patient records as being on antiretroviral treatment (ART) had an RDT test (Determine) run at the clinic; and, in each case, the result recorded on the HIV antenatal surveillance form was HIV-negative. Previous reports have suggested that ART reduces antibody production and can cause sero-reversion (2-4). To explore this further, we ran RDT tests (Determine and Chembio) in parallel at the BRTI laboratory on the left-over DBS specimens for these 7 cases and for an additional 59 known-positive (confirmed HIV-positive with the DNA test) pregnant women who were recorded on the HIV antenatal surveillance form as being on ART but, for which – as recommended in Zimbabwe Ministry of Health and Childcare guidelines – RDT tests were not done at the clinic during the current antenatal check-up. In all 7 original cases, the Determine and Chembio tests both gave sero-reactive results; which could indicate recording errors on the HIV antenatal clinic surveillance forms. For the additional 59 cases, the Determine and Chembio tests produced HIV-positive results in 49 (83.1%) and 56 (94.9%) cases, respectively. There were no cases for which the Determine test result was negative and the Chembio test result was positive.

**Syphilis tests**

For syphilis testing, the SD Bioline Syphilis 3.0 rapid test kit (5) was used to detect *Treponema pallidum* antibodies. In seropositive cases, the remnant of the sample was sent to a district or provincial laboratory for an Rapid Plasma Reagin (RPR) test to establish whether the infection was active or a previously treated infection (6). Syphilis RPR results were returned to the health facility to provide to the client and the result was entered on the ANC surveillance form.

**Malaria tests**

Filter paper dry blood spot (DBS) samples were subjected to DNA extraction using the Chelex method (7). Of the DNA extracts, 2µl was used as template in a 25µl reaction for a Plasmodium species differentiation PCR assay targeting the small sub-unit ribosomal RNA (SSU rRNA) gene of the malaria parasite (8). PCR product (5µl) was analysed by electrophoresis on 2% agarose gel and positives visualized as bands under UV transillumination. All *Plasmodium falciparum* positives were further confirmed by a separate nested PCR targeting the *P. falciparum* dihydrofolate reductase-thymidylate synthetase (DHFR-TS) gene (9).

**2. │ DATA ANALYSIS METHODS**

**Calculations of PPA and NPA adjusted for results from pro-viral DNA tests**

To obtain PPA and NPA measurements based on the quality assurance HIV testing results adjusted for the results of the DNA tests, the results of the DNA tests were taken to be the gold standard. Then it was assumed that, for each combination of antenatal RDT test (or known-positive clinic record status) and laboratory-based ELISA and Innolia test results, the proportion with errors found in the sample of cases tested with the DNA test was representative of the proportion for all cases with the same pattern of antenatal and laboratory results. These calculations are shown for PPA, NPA and known-positive cases in Table S2, Table S3 and Table S4 respectively.

For pregnant women, 45.3% (36/86, Table S1) of the cases with HIV-positive results in the testing done at the central laboratory and HIV-negative in the RDT tests done at the antenatal clinics yielded HIV-negative results when tested with the DNA test. Similarly, 51.6% (16/31) and 95.0% (19/20) of the cases with HIV-negative results in the ELISA or Innolia tests, respectively, done at the central laboratory that had HIV-positive results in the RDT tests done at the antenatal clinics yielded HIV-positive results when tested with the DNA test. If the same proportions with errors occurred in the cases that either were not selected for the DNA test (due to funding constraints) or for which the DBS specimens could not be located, the PPA for pregnant women increases from 82.43% to 89.97% (Table S2), and the NPA increases from 99.6% to 99.9% (Table S3). Fewer laboratory test errors were detected for male partners and the increases in PPA and NPA were smaller.

Similar corrections were made to the PPA/NPA estimates used in the analyses of PPA/NPA in pregnant women by socio-demographic characteristics and of the factors that could have contributed to RDT misclassification errors (Tables 2, 3, S4 & S5). Characteristic-specific adjustments were made for differences in proportions of laboratory test errors only when the sample sizes for which DNA tests were done exceeded 10 cases in both groups (i.e. the groups where the characteristic was present or absent). This was the case for sub-site/main site (present: 52.0%; absent: 45.7%), excess humidity in the HIV testing room (30.8%; 65.4%), and pregnancies in the first trimester (53.3%; 43.5%).

Multivariable analysis of possible sources of false-negative RDT results adjusted for the results of the pro-viral DNA tests was not conducted because DNA tests were only done for sub-samples of cases. However, for exploratory purposes, a multivariable logistic regression analysis was conducted using the unadjusted quality assurance laboratory test results as the gold standard for true HIV infection status. A brief summary of the findings is reported in the Results section below.

**False-HIV-negative RDT test results measured in known-positive cases confirmed by DNA tests**

For known-positive cases, false-negativity rate estimates for the main screening (Determine) and confirmatory (Chembio) RDT tests used at ANCs in Zimbabwe were calculated separately for samples of DNA test-positive cases with HIV-negative and HIV-positive initial laboratory test results. Overall estimates were produced by applying weights for the proportions of the total number of confirmed known-positive cases that had HIV-negative and HIV-positive laboratory results.

**3. │ SUPPLEMENTARY RESULTS**

**Sources of false-HIV-negative RDT errors: multivariable analysis using unadjusted quality assurance data**

In an exploratory multivariable logistic regression analysis of factors hypothesised to be associated with lower PPA (N=1235), storeroom temperature having exceeded the manufacturer’s recommended maximum for the main screening test (Determine) was associated with reduced PPA (aOR=0.50; 95%CI, 0.27 to 0.94). PPA was reduced in women receiving an HIV test in the first trimester of their pregnancy (aOR=0.57; 95%CI, 0.41 to 0.79). Reported adherence to the national HIV testing algorithm and implementation of EQA procedures were also both associated with lower PPA (algorithm adherence: aOR=0.16; 95%CI, 0.05 to 0.51; EQA implementation: aOR=0.69; 95%CI, 0.48 to 0.99). These findings should be treated with caution as the level of misclassification errors in the laboratory results, estimated using the DNA test results, differed for some factors according to whether the factor was present or absent.

**Levels and sources of false-HIV-positive RDT errors**

High levels of NPA were found in females and males in 2017 (Table S5). NPA was lower in Matabeleland South and Mashonaland East than in other provinces, in peri-urban areas than in urban and rural areas, in sub-sites than in main surveillance sites, in 35-54 year-olds than in other age-groups, and in women testing alone compared to those testing as a couple; however, these differences were all small. NPA also varied very little according to the presence and absence of the hypothesised sources of misclassification errors (Table S6). Prior to adjustment for errors identified in the original laboratory quality assurance testing, false-positive cases were found in 2.4% of cases (7/292) where there was non-compliance with Zimbabwe’s national HIV testing algorithm compared with 0.4% of cases (61/16884) where there was compliance with the algorithm.

**Temperature and humidity at the time when RDTs were performed in the Manicaland ANC surveillance sites**

In addition to the data on temperature and humidity collected in the HIV testing rooms and in the health centre storerooms (see Table 3 and Table S6), similar data were collected on the Smartphone camera application at the time each RDT was performed in the seven ANC surveillance sites where this application was used in Manicaland. The temperature exceeded the manufacturer’s maximum recommended level for HIV test results (30^o^C) in 1.9% (22/1184) of cases. The humidity exceeded the manufacturer’s maximum recommended level (60%) in 31.5% (373/1184) of cases. The effect of humidity on RDT can be limited by the storage of the tests in sealed pouches; but the Determine screening test may be more vulnerable as these are stored in a pouch when stored in bulk.

**References**

1. Cepheid. Xpert HIV-1 Qual Sunnyvale, California, USA2019 [Available from: <https://www.cepheid.com/uk/cepheid-solutions/clinical-ivd-tests/virology/xpert-hiv-1-qual>.

2. Jaspard M, Le Moal G, Saberan-Roncato M, Plainchamp D, Langois A, Camps P, et al. Finger-stick whole blood HIV-1/-2 home-use tests are more sensitive than oral fluid-based in-home HIV tests. Public Library of Science One. 2014;9(6):e101148.

3. O’Connell RJ, Merritt TM, Malia JA, VanCott TC, Dolan MJ, Zahwa H, et al. Performance of the OraQuick rapid antibody test for diagnosis of HIV-1 infection in patients with various levels of exposure to highly active antiretroviral therapy. Journal of Clinical Microbiology. 2003;41(5):2153-5.

4. Kufa T, Kharsany ABM, Cawood C, Khanyile D, Lewis L, Grobler AC, et al. Misdiagnosis of HIV infection during a South African community-based survey: implications for rapid HIV testing. Journal of the International AIDS Society. 2017;20(Supplement 6):35-43.

5. Abbott. SD Bioline Syphilis 3.0: one step syphilis anitibody test: Abbott; 2019 [Available from: <https://www.alere.com/en/home/product-details/sd-bioline-syphilis-test.html>.

6. Zimbabwe Ministry of Health and Child Care. HIV antenatal clinic surveillance using PMTCT program data with additional quality monitoring and strengthening in Zimbabwe: study protocol. Harare: Zimbabwe Ministry of Helath and Child Care; 2015.

7. Kain KC, Lanar DE. Determination of genetic variation within *Plasmodium falciparum* by using enzymatically amplified DNA from filter paper disks impregnated with whole blood. Journal of Clinical Microbiology. 1991;29:1171-4.

8. Komaki-Yasuda K, Vincent JP, Nakatsu M, Kato Y, Ohmagari N, Kano S. A novel PCR-based system for the detection of four species of human malaria parasites and Plasmodium knowlesi. Public Library of Science One. 2018;1(1):e0191886.

9. Mharakurwa S, Simoloka C, Thuma PE, Shiff CJ, Sullivan DJ. PCR detection of *Plasmodium falciparum i*n human urine and saliva samples. Malaria Journal. 2006;5:103.
